# A strategy for quality evaluation of salt-treated Apocyni Veneti Folium and discovery of efficacy-associated markers by fingerprint-activity relationship modeling

**DOI:** 10.1038/s41598-019-52963-3

**Published:** 2019-11-13

**Authors:** Cuihua Chen, Jiali Chen, Jingjing Shi, Shuyu Chen, Hui Zhao, Ying Yan, Yucui Jiang, Ling Gu, Feiyan Chen, Xunhong Liu

**Affiliations:** 1 College of Pharmacy, Nanjing University of Chinese Medicine, Nanjing, 210023 China; 2 School of Basic Medicine, Nanjing University of Chinese Medicine, Nanjing, 210023 China; 3 Collaborative Innovation Center of Chinese Medicinal Resources Industrialization, Nanjing, 210023 China; 4 National and Local Collaborative Engineering Center of Chinese Medicinal Resources Industrialization and Formulae Innovative Medicine, Nanjing, 210023 China

**Keywords:** Salt, Secondary metabolism, Mass spectrometry

## Abstract

In this study, a fingerprint-activity relationship between chemical fingerprints and hepatoprotective activity was established to evaluate the quality of salt-treated Apocyni Veneti Folium (AVF). Characteristic fingerprints of AVF samples exposed to different concentrations of salt were generated by ultrafast liquid chromatography tandem triple time-of-flight mass/mass spectrometry (UFLC-Triple TOF-MS/MS), and a similarity analysis was performed based on common characteristic peaks by hierarchical clustering analysis (HCA). Then, the hepatoprotective activity of AVF against CCl_4_-induced acute liver damage in mice was investigated by assessing biochemical markers and histopathology, which showed that a high dose of AVF exposed to low levels of salt stress produced a marked amelioration of hepatic damage compared with the other salt-treated AVF. Finally, fingerprint-activity relationship modeling, which was capable of discovering the bioactive markers used in the quality evaluation, was investigated by the chemical fingerprints and the hepatoprotective activities utilizing multivariate statistical analysis, gray correlation analysis (GCA) and bivariate correlation analysis (BCA). The results showed that the accumulation of polyphenols, such as flavonoids and phenolic acids, in AVF subjected to low levels of salt stress could result in the effective scavenging of free radicals. Therefore, the present study may provide a powerful strategy to holistically evaluate the quality of salt-treated AVF in combination with chemical fingerprint and bioactivity evaluation.

## Introduction

*Apocynum venetum* L., as one of the known medicinal halophytes, has attracted much attention in terms of its antioxidant properties associated with human well-being. This plant grows widely in China, especially in a variety of saline habitats, and therefore, it is characterized by a high physiological plasticity for salt tolerance. Salinity has adverse effects on all aspects of plant health by activating salinity-induced molecular networks associated with salt stress perception, ion and osmotic homeostasis, and regulation of stress-related genes, proteins and metabolic pathways^1,2^. However, halophytes develop a robust and sophisticated protective system and accumulate secondary metabolites under appropriate levels of salt stress, which may be responsible for maintaining homeostasis and protecting against excessive reactive oxygen species-induced oxidative stress^3^.

Apocyni Veneti Folium (AVF) contains abundant bioactive compounds, including the main and prominent antioxidative constituents of phenolic acid and flavonoids^4^. Modern pharmacological studies have demonstrated that AVF has anti-hypertension, anti-depressant, hepatoprotection, anti-anxiety, antioxidation and diuretic functions^5,6^. In the Chinese Pharmacopoeia (2015 edition), hyperoside, a stable active ingredient of AVF occurring at a high concentration, was selected as the marker for the quality control analysis of AVF^7^. In fact, most published reports quantify one or a limited number of components to achieve quality evaluation^8,9^. However, considering the fluctuations in chemical compositions and contents on the basis of many factors, such as abiotic stress, cultivation region and harvest time, it is difficult to achieve consistent quality in medicinal herbs^3^. Furthermore, it is generally recognized that Chinese medicines exert therapeutic efficacies holistically through a ‘multicomponent, multitargeted, and multipathway’ mode^10^. In addition, as mentioned above, AVF is composed of many bioactive components, and its therapeutic effects are not confined to the individual or simple effects of a single bioactive component. Therefore, it is necessary to establish a truly meaningful protocol to effectively and systematically achieve quality control of this plant.

The fingerprint test proved to be a useful tool in assessing the chemical consistency of traditional Chinese medicine (TCM) and has been widely accepted by the World Health Organization (WHO), China Food and Drug Administration (CFDA), the United States Food and Drug Administration (FDA), and European Medicines Agency (EMEA). Several chromatographic techniques, including liquid chromatography (LC)^11^, gas chromatography (GC)^12^, thin-layer chromatography (TLC)^13^, and high-performance capillary electrophoresis (HPCE)^14^, have been used to construct chemical fingerprints of AVF, which has allowed researchers to visualize and identify as many components of the plant as possible. Among these techniques, a high-performance liquid chromatography (HPLC) system is typically employed to establish the chemical fingerprints and quality assessment of AVF because of its ease of operation, high selectivity, and accuracy^9,11,15^. However, limits of this HPLC system include the identification of unknown components. Accurate masses and molecular formulae of untargeted compounds acquired from LC coupled with mass spectrometry/mass spectrometry (LC-MS/MS) have been used to predict and find such components^5,16,17^. In particular, triple time-of-flight mass spectrometry/mass spectrometry (Triple TOF-MS/MS) is considered to be the first accurate, high-throughput and high-resolution system of its kind and operates by means of information-dependent acquisition^18,19^. Therefore, the ultrafast liquid chromatography (UFLC)-Triple TOF-MS/MS system could be introduced to obtain AVF chemical fingerprints. Although similarity analysis of chromatographic fingerprints can directly reflect whether an analyzed sample is chemically similar to others, it alone cannot assess quality consistency. Many studies have proven that samples with high similarity values do not always exhibit the expected equivalent efficacies^20,21^. Hence, fingerprint-activity relationship modeling correlating major components to bioactivity is meaningful to achieve quality consistency and discover bioactive markers^22–25^.

Liver injury induced by carbon tetrachloride (CCl_4_) is one of the most widely used experimental models^26^. Increased serum alanine aminotransferase (ALT), aspartate aminotransferase (AST), and hepatic malondialdehyde (MDA) contents along with decreased superoxide dismutase (SOD), catalase (CAT) and peroxidase (POD) activities demonstrate hepatotoxicity induced by CCl_4_ in mice^27^. Many published studies have indicated that natural substances, such as phenolic acids and flavonoids from plants, exhibit strong antioxidative activity that could act against CCl_4_-induced liver damage^28,29^. References have shown that AVF has protective effects against CCl_4_-induced acute liver injury due to its antioxidative, anti-inflammatory and immunomodulatory constituents^6^.

The main objectives of the present study are thus to chemically and biologically characterize the quality of salt-stressed AVF by fingerprint-activity relationship modeling and discover efficacy-associated bioactive markers. First, the chromatogram fingerprints of AVF exposed to salt stress were established by UFLC-Triple TOF-MS/MS. Then, hierarchical clustering analysis (HCA) was used to discriminate fingerprints based on common characteristic peaks to support the chemical consistency of AVF. Second, CCl_4_-induced acute liver damage in mice was selected to evaluate the consistency of bioactivity according to the quantitative parameters of enzyme activities and histopathological assessment *in vivo*. Third, the fingerprint-activity relationship between common characteristic peaks and efficacy was modeled using multivariate statistical analysis, gray correlation analysis (GCA) and bivariate correlation analysis (BCA) to assess the quality of AVF in response to salt,  and screen out the efficacy-associated bioactive markers and the underlying biological pathways.

## Materials and Methods

### Chemicals and materials

The diagnostic kits for alanine aminotransferase (ALT; serial NO., C009-3-1), aspartate aminotransferase (AST; serial NO., C010-3-1), malondialdehyde (MDA; serial NO., A003-1-2), superoxide dismutase (SOD; serial NO., A001-1-2), catalase (CAT; serial NO., A007-2-1), peroxidase (POD; serial NO., A084-1-1) and protein (serial NO., W042) were purchased from the Institute of Biological Engineering of Nanjing Jiancheng (Nanjing, China). References of silymarin (UV ≥80%), hyperoside and quercetin were obtained from Shanghai Yuanye Biotechnology Co., Ltd. (Shanghai, China); isoquercitrin and chlorogenic acid were purchased from Baoji Chenguang Biotechnology Co., Ltd. (Baoji, China). Cryptochlorogenic acid was acquired from Chengdu Chroma Biotechnology (Chengdu, China). HPLC-grade acetonitrile was purchased from Merck (Darmstadt, Germany). CCl_4_ and olive oil were purchased from Aladdin (Shanghai, China).

### Sample preparation

Salt stress experiments have been described in detail in our previous papers^3^. Briefly, 4 salt treatment concentrations were used: 0 (control), 100 (low stress), 200 (medium stress) and 300 (high stress) mM NaCl treatments were designed with 3 replicates and 3 pots per replicate. After 12 h of the last salt treatment experiment, AVF samples were harvested and dried at room temperature. Naturally dried, salt-treated samples were powdered and passed through a 60 mesh sieve. Four groups of dried samples (20 g) were extracted twice with 200 mL of 70% (v/v) ethanol for 2 h under reflux followed by centrifugation at 3,500 rpm for 10 min, and the supernatant was collected. After rotary evaporation and freeze-drying to a powder, a small portion was redissolved in 70% ethanol to form a 1 mL solution containing 0.05 g of AVF, and the solution was centrifuged at 12,000 rpm for 15 min. Then, the supernatant was stored at 4 °C and filtered through a 0.22 μm membrane before being subjected to UFLC-Triple TOF-MS/MS analysis. The rest of the freeze-dried powder was redissolved with 0.5% sodium carboxymethyl cellulose (CMC-Na) to form a 1 mL solution containing 0.5 g of AVF for the animal experiments. Mixed reference standard solutions were prepared by accurately weighing corresponding amounts of reference compounds dissolved in 70% ethanol to obtain a final concentration of approximately 1 μg/mL.

### UFLC-Triple TOF-MS/MS fingerprint analysis and clustering analysis

MS data were recorded by a high-resolution quadrupole time-of-flight mass spectrometer (Triple TOF^TM^ 5600 System-MS/MS, AB Sciex, Framingham, MA, USA) equipped with an electrospray ionization (ESI) source. Chemical fingerprints were obtained and peak identification was performed via the UFLC-Triple TOF-MS/MS system. An XBridge^®^ C_18_ column (100 mm × 4.6 mm, 3.5 μm) was used for the analysis. The mobile phase consisted of water containing 0.1% formic acid (A) and acetonitrile containing 0.1% formic acid (B). The samples were eluted using a linear gradient program as follows: 0–3 min, 5% B; 3–8 min, 5–18% B; 8–12 min, 18–20% B; 12–15 min, 20% B; 15–17 min, 20–60% B; 17–18 min, 60–80% B; 18–18.5 min, 80–5% B; and 18.5–22.1 min, 5% B. The flow rate was set at 0.8 mL min^−1^ with the column maintained at 30 °C, and the injection volume was 5 μL. Runs under the positive and negative ion modes were set separately as follows: nebulizer pressure, 55 psi; drying gas pressure, 55 psi; curtain gas pressure, 40 psi; source temperature, 550 °C; and capillary voltage, 5500 V and −4500 V, respectively, for the runs under positive and negative ion modes. Data were acquired for each sample from 50 to 1,500 Da, and dynamic range enhancement was applied throughout the MS experiment to ensure accurate mass measurement.

Based on the established methods, precision and repeatability were assessed independently by successively analyzing 6 injections of sample 1 solution and 5 replicates of 100 mM NaCl-stressed AVF solution, respectively. Stability was assessed by analyzing one sample over a 48 h time period (0, 4, 8, 12, 24 and 48 h). “Chinese traditional medicine chromatographic fingerprint similarity evaluation system 2004, A Edition” was used to correct the retention time of each peak. The peak area was processed by equalization to obtain quantitative data. The reference atlas was generated with the median method from a general comparison of chromatograms obtained for the 12 samples. Similarity between the reference fingerprints and various chromatograms was determined by the software. Common characteristic peaks were identified by comparison with the reference compounds, while for compounds without a standard reference, the characteristic peaks were inferred using PeakView 1.2 software (AB SCIEX, USA), references on AVF^5,6,9^ and online resources, such as HMDB (http://www.hmdb.ca/), SciFinder (https://www.cas.org/products/scifinder) and PubChem (https://pubchem.ncbi.nlm.nih.gov/) through comparing MS/MS fragment ions.

HCA was introduced to cluster samples based on common characteristic peaks by Cluster 3.0 and Java Treeview 3.0. Clustering was based on the Euclidean distance coefficient and average linkage method. The mean values of all parameters were taken from the measurements of three samples, and every sample had three replicates from which the standard deviations were calculated.

### Assessment of liver function

SPF-grade ICR male mice (weighing 18–22 g) were obtained from Qinglong Mountain Animal Breeding Farm Limited Company (Jiangning District, Nanjing, China). All animals were maintained under standard laboratory conditions (temperature 22 ± 2 °C, relative humidity 50 ± 10%) with dark and light cycles (12/12 h). The mice were acclimatized to laboratory conditions for 3 days before the commencement of the experiment. All animal procedures were approved by the IACUC (Institutional Animal Care and Use Committee) of Nanjing University of Traditional Chinese Medicine and carried out in accordance with the Guidelines of Accommodation and Care for Animals formulated by the Chinese Convention for the Protection of Vertebrate Animals used for Experimental and Other Scientific Purposes. All efforts were made to minimize animal suffering as well as to reduce the number of animals required for experimentation.

Mice were randomly divided into five groups of 11 animals each. The experimental process is summarized in Table S1. In the control and CCl_4_-intoxicated groups, animals were given a single dose of 0.5% CMC-Na orally using a gavage daily for 14 days. A positive control group was administered silymarin (100 mg/kg body weight (bw)) as a reference drug. The low dose (0.3 g/kg bw) and high-dose (3 g/kg bw) groups were given different treatments of AVF exposed to various levels of salt stress, named L0, L100, L200, L300, H0, H100, H200 and H300 at 0.1 mL/10 g bw and a 0.2 mL volume. After 2 h of the last treatment, the control group was given olive oil (10 mL/kg), while the other groups were simultaneously injected intraperitoneally (i.p.) with 10 mL/kg of 0.3% (v/v) CCl_4_ in olive oil. Then, all animals were fasted for 16 h and anesthetized using sodium pentobarbital (1% in physiological saline, 50 mg/kg; i.p.). Blood was collected immediately from the fundus vein and kept at 4 °C. After death, the liver was removed and placed in chilled saline solution, dried with filter paper and excised into two portions. One part was frozen quickly using liquid nitrogen and stored at −80 °C until the preparation of hepatic homogenates for the determination of biochemical parameters, while the other portion was preserved in 10% formalin for histopathological investigations.

During the whole experiment, the general conditions and bw of mice were monitored at 0, 7 and 14 days. For biochemical analysis, assays were conducted using kits obtained from the Institute of Biological Engineering of Nanjing Jiancheng (Nanjing, China) according to the manufacturer’s protocol. Blood was centrifuged at 3,000 rpm for 10 min at 4 °C, and then the serum levels of ALT and AST were determined by colorimetry^30^. Liver tissues (0.1 g) were excised and homogenized using a homogenizer. The sample livers were homogenized on ice with a 9× volume of homogenate prepared by precooled saline solution (0.9%) and centrifuged at 3,500 rpm for 15 min. The tissue homogenates were inspected for the determination of total soluble proteins by the Bradford method, and the calibration curve was prepared using solutions of bovine serum albumin (BSA)^31^. Absorbance was recorded at 562 nm via a UV-visible spectrophotometer (Shanghai Spectrum Instrument Co., Ltd.). The MDA content was detected by the thiobarbituric acid method^32^; SOD activity was measured at 550 nm following the nitro-blue tetrazolium method; and the basis for the evaluation CAT activity was decomposition of H_2_O_2_ to less reactive molecules, which was recorded at 240 nm and assessed by following the kit instructional protocol^3,33,34^. POD activity was estimated on the basis of guaiacol peroxidation measured at 420 nm^35,36^.

Histopathological observation was performed as follows: liver tissues preserved in 10% neutral formalin solution for at least 24 h were embedded in paraffin and cut into 5 μm thin sections, deparaffinized, dehydrated, and stained with hematoxylin-eosin for the estimation of hepatocyte necrosis and vacuolization. Histopathological changes and injuries were observed under light microscopy (Stereo Investigator, MBF Bioscience), and photographs were captured at 400× magnification.

### Fingerprint-effect relationship modeling

GCA and BCA were performed to assess the fingerprint-activity relationship model with Excel 2010 for Windows 10 and SPSS 17.0 statistical software (SPSS Statistics 17 for Windows 10, SPSS Inc.), respectively. The correlation coefficient (*ξ*) derived from the GCA represents the degree to which the correlation factor sequence is close to the behavioral feature. The higher the value of *ξ* is, the closer the sequence of associated factors to the behavioral characteristics. Detailed steps can be found in the experimental section of the supporting information. The basic idea of BCA is to study the correlation between two sets of variations^37^, and BCA is widely used in the quality evaluation of TCMs^38^. Fingerprint-activity relationship analysis was performed by transferring common fingerprint peak area and pharmacological test data into SPSS software, and then BCA was used to assess the spectrum-effect relationships between the areas of common characteristic peaks in the fingerprint and the main hepatoprotective parameters. The statistical analysis was performed by one-way ANOVA followed by Duncan’s multiple-range test to compare the means with the significance level set as 0.05 by SPSS 17.0. Biological pathway analysis was constructed based on the bioactive markers according to KEGG and MetaboAnalyst 4.0. Colors varying from yellow to red indicate that the metabolite data have different levels of significance.

## Results and Discussion

The many antioxidative phytochemicals of AVF have been extensively used for countering oxidative stress and oxidation-related impairments. Moreover, a considerable amount of AVF extract compounds have been investigated and confirmed to have hepatoprotective effects^6,39^. Studies have demonstrated that phenolic compounds can prevent CCl_4_-induced lipid peroxidation and hepatotoxicity due to antioxidative activities^40^. Therefore, in this study, fingerprint-activity relationship modeling by UFLC-Triple TOF-MS/MS analysis was performed to assess the quality of AVF exposed to different concentrations of salt and discover bioactive markers associated with efficacy of the AVF *in vivo*.

### Fingerprints and similarity analysis of AVF samples

**Figure 1:**
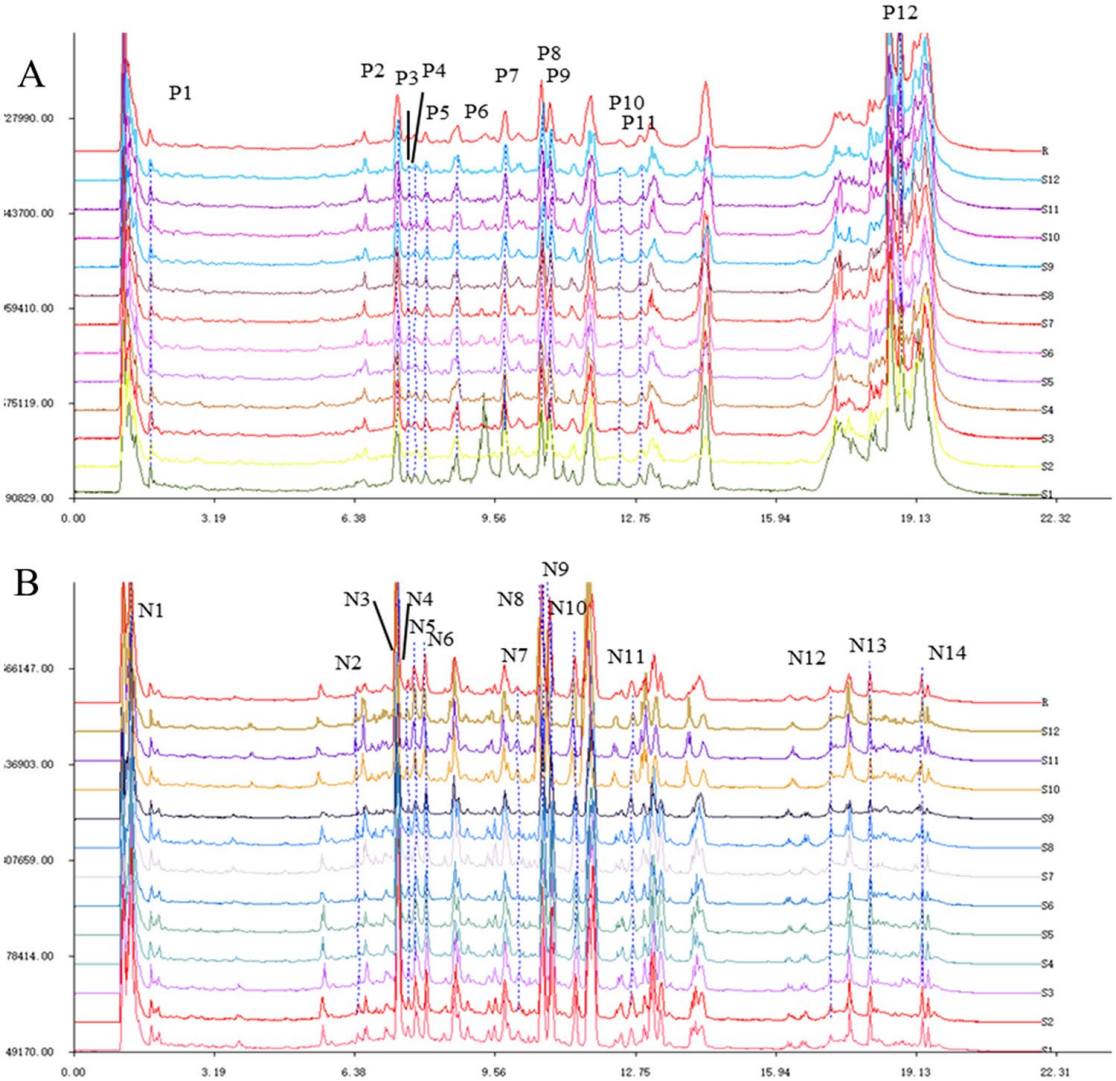
Representative UFLC-Triple TOF-MS/MS fingerprints of AVF exposed to salt stress under positive (**A**) and negative (**B**) ion modes.

The representative base peak chromatograms obtained from the analysis under the positive and negative ion modes of UFLC-Triple TOF-MS/MS for the reference mixture and AVF were obtained under the optimal conditions (Fig. S1). A total of 12 peaks with large areas and good separation that were found in all chromatograms of the samples under the positive ion mode were regarded as “common characteristic peaks”, and they were labeled with P in the fingerprints; 14 common characteristic peaks detected under the negative ion mode, labeled with N and with the corresponding peak and relative peak areas, are displayed in Fig. 1 and Table S2. Thus, the present study established fingerprints of AVF exposed to salt stress. Comparisons were performed of the *t*_R_ and *m/z* values of the reference compounds and MS/MS fragment ions with values from references^5,6,9^ and online databases for the compounds without standard chromatography data, and the information for these peaks is presented in Table 1.

**Table 1:** Identification of the common characteristic peaks of AVF under salt stress.

Peak No.	*t*_R_	Compounds	Molecular formula	*m/z adducted ion*	Fragment ions
P1	1.74	Citric acid/isocitric acid	C_6_H_8_O_7_	193.0348[M + H]^+^	193.0346 175.0243 133.0137
P2	7.34	Chlorogenic acid^*^	C_16_H_18_O_9_	355.0956[M + H]^+^	355.1034 163.0388
P3	7.59	Unknown	Unknown	506.2806[M + H]^+^	506.2872 327.2029 133.0869
P4	7.74	Cryptochlorogenic acid^*^	C_16_H_18_O_9_	355.1031[M + H]^+^	355.103
P5	7.98	Procyanidin B2	C_30_H_26_O_12_	579.1497[M + H]^+^	579.1519 395.132
P6	8.71	Glaucolide B	C_21_H_26_O_10_	441.1166[M + H]^+^	441.1155 207.0665 149.0586
P7	9.67	Quercetin-3′-glucuronide	C_21_H_20_O_13_	481.0968[M + H]^+^	319.0479
P8	10.63	Hyperoside^*^	C_21_H_20_O_12_	465.1039[M + H]^+^	465.1038 303.0511
P9	10.82	Isoquercitrin^*^	C_21_H_20_O_12_	465.1054 M + H]^+^	465.1054 303.0535
P10	12.39	Acetylated isoquercitrin	C_23_H_22_O_13_	507.1140[M + H]^+^	303.0454 507.113
P11	12.87	Quercetin 3-*O*-(6″-*O*-malonyl)-β-D-glucoside	C_24_H_22_O_15_	551.1035[M + H]^+^	551.1036 303.0510 163.1328
P12	18.77	Allamandin	C_15_H_16_O_7_	309.2075[M + H]^+^	291.1953 273.1845 119.0869 79.0560
N1	1.28	Shikimic acid	C_7_H_10_O_5_	173.0464[M − H]^−^	173.0450 93.0348
N2	6.43	Unknown	Unknown	707.1892[M − H]^−^	707.1954 353.089 191.0589
N3	7.34	Chlorogenic acid^*^	C_16_H_18_O_9_	353.0889[M − H]^−^	85.0320 191.0576
N4	7.58	Unknown	Unknown	691.1916[M − H]^−^	691.1916 451.2208 162.8429 119.0521 93.0358
N5	7.75	Cryptochlorogenic acid	C_16_H_18_O_9_	353.0889[M − H]^−^	135.0473 179.0372 191.0584 353.0905
N6	7.98	Procyanidin B2	C_30_H_26_O_12_	577.1391[M − H]^−^	289.0728 407.0795 577.1383
N7	10.09	Unknown	Unknown	461.1694[M − H]^−^	415.1666 191.0588 149.0478
N8	10.62	Hyperoside^*^	C_21_H_20_O_12_	463.0882[M − H]^−^	301.0391 463.0923
N9	10.82	Isoquercitrin^*^	C_21_H_20_O_12_	463.0885[M − H]^−^	463.0902 301.0378 151.0055
N10	11.38	Quercetin 3-*O*-(6″-*O*-malonyl)-β-D -galactoside	C_24_H_22_O_15_	549.0926[M − H]^−^	505.1042 463.0896 301.0377 300.0301
N11	12.67	Quercetin 3-*O*-(6″-*O*-malonyl)-β-D-glucoside	C_24_H_22_O_15_	549.0916[M − H]^−^	505.1051 301.0377 300.0301
N12	17.68	Quercetin^*^	C_15_H_10_O_7_	301.0364[M − H]^−^	151.0055 179.0007 273.0428 301.0382
N13	18.08	Kaempferol*	C_15_H_10_O_6_	285.0417[M − H]^−^	93.0363 285.0436
N14	19.25	Hyperforin	C_35_H_52_O_4_	535.3787[M − H]^−^	535.3778

* Indicates that compounds were identified according to the reference substances.

The validation of the method showed that the experimental precision was less than 0.5% for *t*_R_ and 0.9% for the peak area of common characteristic peaks. The method stability was less than 0.29% for *t*_R_ and 1.9% for the peak area of common characteristic peaks. The repeatability was less than 0.3% for *t*_R_ and 1.8% for the area of common characteristic peaks. The similarity values between the reference standard and chromatographic fingerprints for 12 samples ranged from 0.7 to 0.901 and from 0.839 to 0.962 under positive and negative ion modes, respectively. The results of precision, stability and repeatability analysis suggested the validity and suitability of the optimized method for analyzing samples.

**Figure 2:**
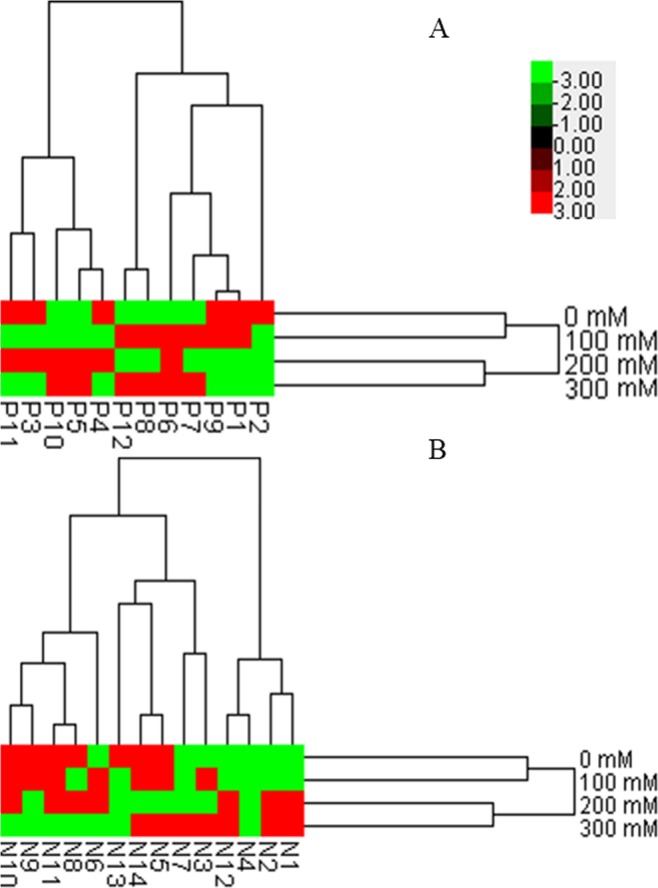
Hierarchical clustering analysis based on the data of common characteristic peaks of AVF subjected to salt stress under positive (**A**) and negative (**B**) ion modes.

A heat map derived from HCA graphically displays the changes in the accumulation of common characteristic compounds under salinity stress (Fig. 2) and the clustering of samples. Clear differentiation was observed in the positive and negative ion mode results, revealing that AVF samples could be classified into two clusters, that is, the 0/100 and 200/300 mM salt-treated samples formed clusters that were clearly separate from each other. The assessment of clustering based on common characteristic peaks was consistent with the similarity analysis both in the positive and negative ion modes and was also in agreement with our previous report on the metabolitesof AVF in response to salt stress^3^. A clear stress-induced trajectory for metabolomic changes was evident, indicating the dose-dependent responses of salt-stressed AVF. Thus, HCA could qualitatively compare salt-treated samples and effectively separate these samples.

### Hepatoprotective activity

**Figure 3:**
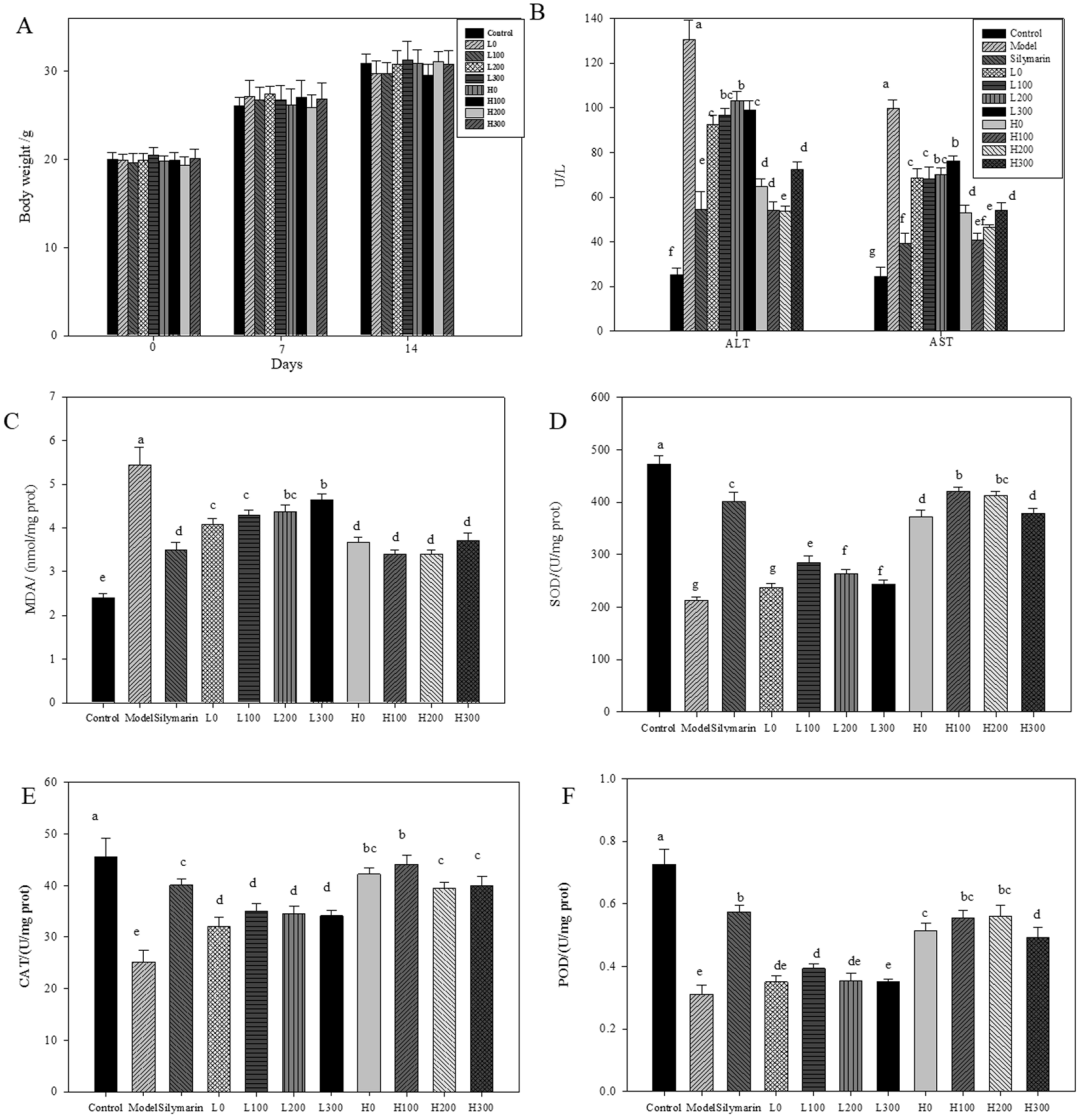
Effects of AVF extract on body weight (**A**), ALT and AST activities (**B**), hepatic MDA content (**C**) and activities of antioxidant enzymes SOD (**D**), CAT (**E**) and POD (**F**) after CCl_4_ treatment in mice. Data are the mean ± SD (n = 6). Different letters following values (a, b, c, d, e, f and g) in the same row indicate significant differences among salt treatments using Duncan’s multiple-range test at *p* < 0.05.

The function of AVF extract in protecting against CCl_4_-induced hepatotoxicity was investigated to study its medicinal efficacy. Bw is a significant indicator of the negative effects of xenobiotics and is a determinant constraint in toxicity analysis. In the present study, no significant changes were observed in the coadministration of AVF to CCl_4_-treated mice (Fig. 3A). The outcomes were in agreement with the previous findings on AVF^40^.

Metabolic products of CCl_4_ not only affect the permeability of membranes, resulting in an increase in serum aminotransferase and lipid peroxidation but also decrease the activities of antioxidative enzymes^41^. ALT and AST are indications of hepatic impairment induced by CCl_4_ and are usually considered subtle markers of liver function. The hepatoprotective effects of AVF on the serum ALT and AST activities are shown in Fig. 3B and Table S3. In the CCl_4_-intoxicated model group, a significant increase in the ALT and AST activities was shown in the livers exposed to CCl_4_ toxicity, with values up to 130.67 and 99.77 U/L, respectively, whereas the values of the control group were only 25.4 and 24.63 U/L, respectively. Silymarin, which has been used to treat hepatotoxic diseases in clinical practice for decades^42,43^, significantly inhibited enzyme activities, with the ALT and AST activities reduced to 58.19% and 60.16%, respectively. Administration with a low dose (0.3 g/kg) of AVF stressed by four concentrations of NaCl for 14 days prior to CCl_4_-induced hepatotoxicity significantly decreased ALT activity by 29.16%, 25.89%, 20.97% and 24.16%, respectively, and AST activity by 31.14%, 31.47%, 29.84% and 23.72%, respectively, compared to the model group levels. Administration of a high dose (3 g/kg) of AVF prior to CCl_4_ injury markedly reduced hepatotoxicity, with the aminotransferase activities decreasing by approximately half, indicating promising effects of AVF protection against CCl_4_-induced damage by the regulation of enzymatic expression to a level similar to that of silymarin. Our results were in accordance with other findings in which treatment with a crude methanol extract of medicinal plants restored the levels of liver biochemical markers after CCl_4_ treatment^35^.

Pretreatment with AVF effectively inhibited CCl_4_-induced hepatotoxicity and significantly reduced the level of MDA, which was inversely proportional to the salt dose (Fig. 3C and Table S3). Briefly, an increase was shown in MDA content with increasing salt concentrations. At the same time, coadministration with AVF extract at a high dose significantly inhibited hepatic MDA formation, having nearly the same effect as silymarin, and coincidentally, there was no significant difference between the five groups, indicating that AVF could effectively inhibit MDA formation or eliminate excess free radicals.

SOD, CAT, and POD activities were also investigated to study the efficacy of AVF in the ability to restore and maintain activities in CCl_4_-injured livers (Fig. 3D–F and Table S3). Specifically, SOD catalyzes the dismutation of O_2_^−^ into O_2_ and H_2_O_2_, whereas CAT and POD are responsible for the removal of H_2_O_2_^3,44^. The increased production of free radicals was a major cause of the significantly reduced SOD activity in the model. In the model, SOD, CAT and POD activity was markedly reduced by nearly half compared with the control levels; however, mice administered a low dose of AVF showed varied responses. In detail, SOD activity increased significantly compared with that of the model group except in the L0 group, and the L100 group showed the highest value. The levels of SOD activity induced by salt-stressed AVF from high to low were found with H100, H200, H300 and H0, indicating that low and medium salt levels altered the plant constituents associated with liver-protecting activity. For CAT, no significant difference was shown among any group treated with a low dose of AVF exposed to salt stress, but each group showed higher CAT activity than the model group. In addition, the CAT activity in mice pretreated with watering AVF did not change significantly compared to that of the salt-treated groups at a high dose. This result suggested that salt-stressed AVF was superior to the control and that the low-salt treatment worked better at elevating antioxidant enzyme activity^3^. In addition, POD activity did not show marked changes among the low-dose groups except for L100, which showed significantly increased levels compared to those of the control; however, POD activity increased significantly in the high-dose groups compared with the control group, and POD activity decreased with increasing salt concentrations, revealing the failure of this enzyme to scavenge excessive free radicals.

**Figure 4:**
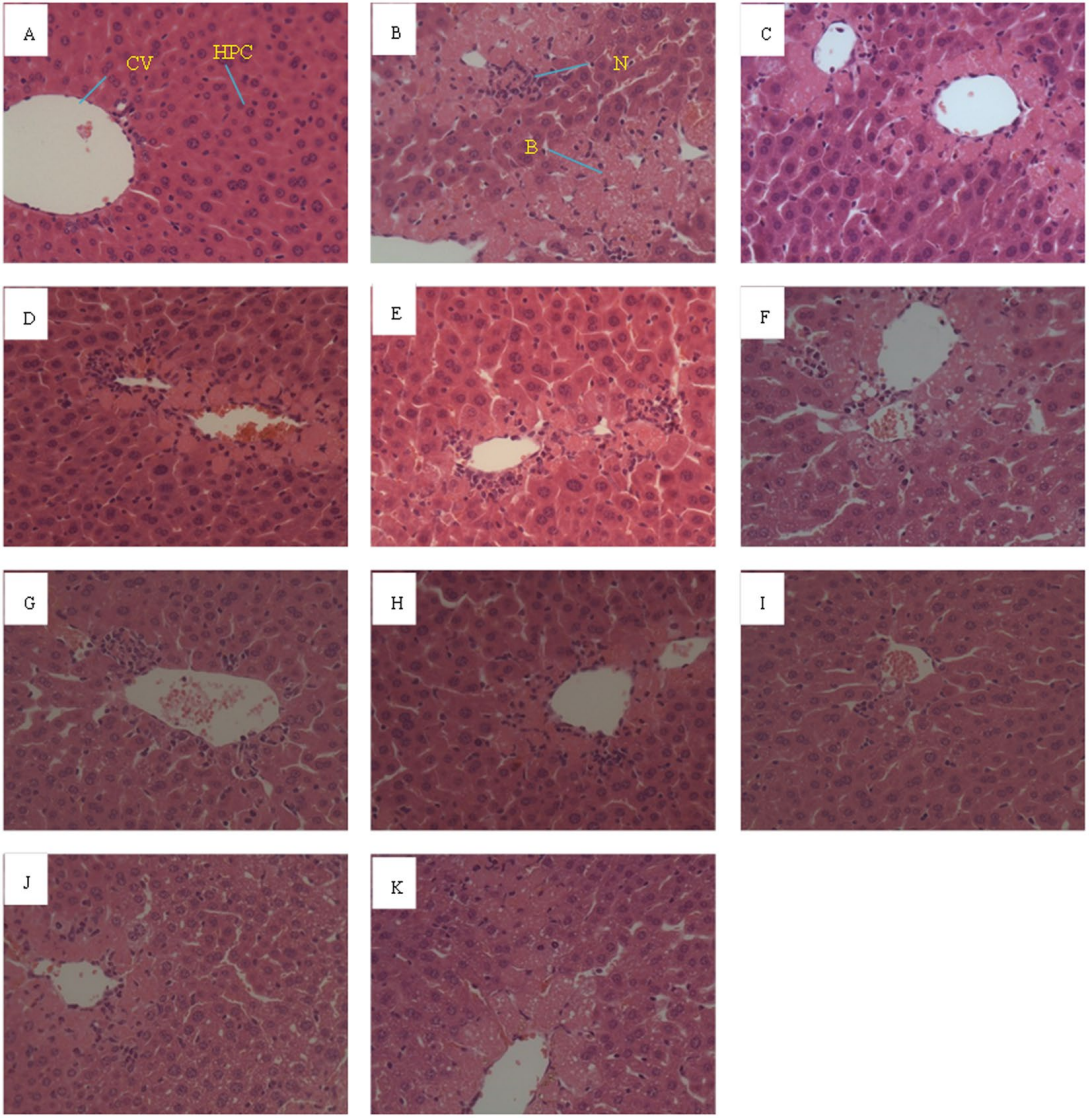
Representative photographs of histological liver damage after CCl_4_ treatment in mice. (**A**) Control group. (**B**) CCl_4_-intoxicated model group. (**C**) Shows mice tissue pretreated with silymarin prior to CCl_4_ treatment. (**D**–**G**) Showed tissue pretreated with 0, 100, 200 and 300 mM salt-stressed AVF (0.3 g/kg) prior to CCl_4_ intoxication, respectively. (**H**–**K**) Were treated with 0, 100, 200 and 300 mM salt-stressed AVF (3 g/kg), respectively, prior to CCl_4_ intoxication.

The protective effects of AVF against CCl_4_-induced hepatotoxicity were further confirmed by histopathological observations. Representative photographs of liver sections stained with hematoxylin-eosin in hepatic tissues under microscopy (400×) are shown in Fig. 4. In the control mice, normal histology was observed, with a well-preserved cytoplasm and prominent nucleus in hepatic cells. In CCl_4_-treated mice, extensive liver injuries were observed in hepatocytes, characterized by hepatocellular degeneration and necrosis around the central vein, inflammatory cell infiltration, ballooning degeneration, and the loss of cellular boundaries. However, coadministration with a low dosage of AVF prior to CCl_4_ treatment reduced the extent of histopathological injury. Furthermore, silymarin and high-dose AVF, especially 100 mM salt-stressed AVF, restored the altered parameters to the control levels. These results were in good agreement with the serum aminotransferase and hepatic antioxidant enzyme activity results, and similar results have been found for *Brachychiton populneus* extract against CCl_4_-induced liver injury^35^. Although the effects of salt-treated AVF manifested differently with respect to liver protection, the liver injury was remarkably ameliorated by pretreatment with H100, resulting in lower hepatocellular degeneration and less hydropic necrosis.

### Fingerprint-activity relationship modeling analysis

The assessment of spectrum-effect relationships represents a powerful tool for the quality control of herbal medicines. However, the key difficulty in the application of spectral relationships to TCMs is how to associate complex chromatographic peaks with pharmacodynamic information^24,45^. Fingerprint-activity relationship modeling of TCM is based on the guidance of TCM theory. The fingerprints and pharmacodynamics of TCMs, which are correlated by chemometric models, establish a comprehensive evaluation system for revealing the material basis of the pharmacodynamics of TCMs^46^. This has been one of the approaches used for predicting active ingredients and performing quality control in natural products. In this study, multivariate statistical analysis was conducted to associate chemical composition with efficacy to evaluate AVF quality and, furthermore, to discover bioactive markers. GCA is often used to reveal quantitative comparisons of trends in dynamically changing systems and is suitable for solving problems with complicated interrelationships between multiple factors and variables^47,48^. GCA also provides a reliable method for the quality evaluation of TCMs. Compared with other analysis methods, such as regression analysis and canonical correlation analysis, GCA has several advantages, such as requiring only a small sample size and a small amount of calculations and providing intuitive results^49,50^. GCA can be used to assess the size of the relevance between the efficacy index and the chromatographic peak and offers a possibility for predicting the active components^51^. BCA is also known as canonical correlation analysis. The basic idea of BCA is to study the correlation between two sets of variations^19,37^, revealing much of the information about them and finding their linear combinations that have the highest correlation^22^. The Pearson correlation coefficient has a clear quantitative meaning and can reflect the magnitude and direction of the linear correlation between variables^52^. Although the outcomes analyzed by GCA and BCA were not completely consistent, there were either synergetic or antagonistic effects among these components^52^.

In the GCA, the correlation coefficient of peaks was between 0.581 and 0.963 (Table 2). Specifically, 7 peaks were closely correlated with efficacy, including peaks P1, P4, P5, N1, N9, N10 and N12, and the top five compounds with large correlation coefficient values for each indicator were selected as markers, so 17 peaks (P1, P2, P3, P4, P5, P8, P10, P11, N1, N3, N4, N6, N8, N9, N10, N11 and N12) were screened out. Furthermore, BCA clearly showed that P10, P12 and N6 were correlated with ALT with the absolute value of the Pearson correlation coefficient (*r*) being greater than 0.5 (Table 3). Similarly, N11 and N14 were negatively correlated with AST; for MDA, the same was true for N6 and P10. In addition, P10 appeared to have a negative correlation with SOD activity, while N9 and N14 were positively corrected with CAT activity and N8 and N11 were positively corrected with POD activity. Therefore, 7 peaks (P10, P12, N6, N8, N9, N11 and N14) were screened out. In other words, these peaks might represent the main hepatoprotective components in the AVF extract, but further study is needed to identify the chemical structures and to confirm their bioactivities. These results will be helpful for the study of AVF and other herbs to search for their effective components. In addition, the relative contents (peak areas) showed that the quality level was highest in samples treated with a low concentration of salt compared to that of other groups. This further implies that the spectrum-effect relationship method is efficient for quality evaluation.

**Table 2:** Gray correlation analysis between fingerprints and the efficacy of AVF exposed to salt stress.

Order	ALT		AST		MDA		SOD		CAT		POD	
	Peak No.	Correlation coefficient(ξ)	Peak No.	Correlation coefficient(ξ)	Peak No.	Correlation coefficient(ξ)	Peak No.	Correlation coefficient(ξ)	Peak No.	Correlation coefficient(ξ)	Peak No.	Correlation coefficient(ξ)
1	P1	0.826	P4	0.902	P5	0.939	P1	0.934	P5	0.948	P2	0.944
2	P12	0.806	P5	0.885	P8	0.921	P2	0.928	P8	0.931	P11	0.936
3	P8	0.791	P8	0.880	P10	0.909	P3	0.894	P11	0.912	P5	0.897
4	P9	0.788	P10	0.857	P4	0.901	P11	0.876	P2	0.877	P1	0.885
5	P5	0.772	P11	0.842	P11	0.890	P5	0.875	P4	0.869	P8	0.883
6	P2	0.768	P2	0.840	P2	0.868	P8	0.873	P10	0.841	P3	0.849
7	P3	0.759	P12	0.825	P12	0.830	P9	0.856	P12	0.828	P9	0.820
8	P10	0.705	P6	0.809	P1	0.823	P10	0.803	P1	0.814	P10	0.818
9	P11	0.701	P1	0.808	P6	0.803	P7	0.783	P3	0.797	P4	0.791
10	P4	0.686	P3	0.782	P3	0.797	P6	0.771	P6	0.789	P7	0.771
11	P6	0.660	P9	0.773	P9	0.780	P4	0.746	P9	0.765	P6	0.765
12	P7	0.581	P7	0.755	P7	0.754	P12	0.739	P7	0.724	P12	0.764
1	N12	0.831	N1	0.914	N1	0.957	N9	0.872	N10	0.948	N10	0.963
2	N3	0.803	N4	0.902	N4	0.923	N10	0.861	N4	0.908	N1	0.946
3	N4	0.776	N12	0.876	N10	0.894	N3	0.835	N1	0.904	N8	0.933
4	N5	0.769	N3	0.866	N12	0.873	N6	0.825	N6	0.893	N11	0.929
5	N11	0.759	N10	0.848	N8	0.869	N11	0.825	N8	0.892	N6	0.911
6	N9	0.752	N6	0.846	N3	0.858	N4	0.807	N11	0.878	N4	0.910
7	N6	0.748	N8	0.819	N6	0.843	N5	0.792	N3	0.858	N9	0.896
8	N7	0.747	N2	0.795	N11	0.829	N1	0.778	N12	0.832	N5	0.870
9	N10	0.743	N11	0.794	N9	0.785	N8	0.777	N9	0.822	N13	0.869
10	N1	0.721	N9	0.767	N2	0.741	N12	0.727	N5	0.722	N3	0.863
11	N13	0.712	N5	0.736	N5	0.739	N14	0.706	N2	0.665	N14	0.830
12	N8	0.658	N7	0.706	N7	0.679	N13	0.639	N7	0.665	N12	0.827
13	N14	0.581	N14	0.679	N14	0.669	N2	0.620	N14	0.638	N7	0.718
14	N2	0.555	N13	0.631	N13	0.623	N7	0.615	N13	0.607	N2	0.590

**Table 3:** Bivariate correlation analysis between fingerprints and the efficacy of AVF exposed to salt stress.

Peak No.	Pearson correlation coefficient (*r*)						Peak No.	Pearson correlation coefficient(*r*)					
	ALT	AST	MDA	SOD	CAT	POD		ALT	AST	MDA	SOD	CAT	POD
P1	−0.130	−0.181	0.077	0.223	0.453	−0.009	N1	−0.207	0.125	−0.190	0.200	−0.339	0.067
P2	−0.045	−0.097	0.109	−0.154	0.413	0.003	N2	0.115	0.103	−0.121	0.052	−0.339	−0.086
P3	0.226	0.304	0.115	−0.196	0.294	−0.360	N3	−0.138	−0.137	−0.220	0.307	0.161	0.173
P4	−0.421	−0.291	−0.072	0.184	−0.301	0.462	N4	−0.207	−0.086	−0.330	0.131	0.052	0.224
P5	−0.066	−0.382	0.275	0.114	0.019	0.092	N5	−0.139	−0.304	−0.213	0.093	0.521	0.194
P6	−0.295	−0.405	−0.366	0.481	0.457	0.403	N6	−0.568	−0.447	−0.538	0.421	0.378	0.433
P7	−0.385	−0.223	−0.217	0.397	0.382	−0.028	N7	0.227	−0.035	−0.085	0.052	0.120	−0.142
P8	−0.131	−0.165	−0.075	0.244	0.396	0.222	N8	−0.455	−0.286	−0.332	0.116	0.400	0.560
P9	−0.146	0.081	−0.072	0.088	0.271	−0.214	N9	−0.436	−0.273	−0.250	0.114	0.656*	0.170
P10	0.703^*^	0.498	0.526	−0.549	−0.400	−0.404	N10	−0.340	−0.207	−0.295	0.075	0.290	0.146
P11	−0.152	−0.114	0.000	−0.097	0.023	0.114	N11	−0.499	−0.631*	−0.405	0.493	0.404	0.575
P12	0.543	0.302	0.114	−0.075	−0.422	−0.274	N12	−0.074	−0.055	−0.426	0.304	−0.287	0.416
							N13	0.240	0.225	0.265	−0.386	0.211	−0.420
							N14	−0.296	−0.524	−0.363	0.254	0.701*	0.251

*p < 0. 05.

### Biomarker identification

Based on the GCA and BCA results, 19 peaks (P1, P2, P3, P4, P5, P8, P10, P11, P12, N1, N3, N4, N6, N8, N9, N10, N11, N12 and N14) were correlated with pharmacodynamic effects. P2/N3, P5/N6, P8/N8 and P11/N11 were identified as the same compound, respectively. Therefore, 15 bioactive markers were obtained, including organic acids (citric acid/ isocitric acid and shikimic acid), flavonoids (quercetin, hyperoside, isoquercitrin, procyanidin B2, quercetin 3-*O*-(6″-*O*-malonyl)-β-D-glucoside, acetylated isoquercitrin, and quercetin 3-*O*-(6″-*O*-malonyl)-β-D-galactoside), terpenoids (allamandin), phenylpropanoids (chlorogenic acid and cryptochlorogenic acid), quinones (hyperforin) and two unknown compounds (P3 and N4). This finding revealed that the defensive properties of AVF might be attributed to the presence of potent antioxidant constituents preventing tissue degeneration by inhibiting lipid peroxidation of the liver.

The metabolites citric acid/isocitric acid and shikimic acid are key metabolic components in the tricarboxylic acid cycle (TCA cycle), and the alteration of these metabolites might disrupt energy metabolism. Citric acid acted as a potential biomarker, showing hepatoprotective effects and contributing directly to the therapeutic effect of *Angelica sinensis*^53^, and shikimic acid was associated with the biosynthesis of phenylpropanoids. Published studies on chlorogenic acid have shown that it plays several important roles, such as displaying hepatoprotective activity by suppressing liver fibrogenesis and carcinogenesis, modulating lipid metabolism and scavenging free radicals^54,55^, while cryptochlorogenic acid isolated from *Artemisia capillaris* possesses potent activity against HBV DNA replication^56^.

The most abundant bioactive flavonoid constituents correlated with hepatoprotective activity were quercetin, procyanidin B2, hyperoside, isoquercitrin, and derivatives of hyperoside/isoquercitrin. The potential mechanism of quercetin-induced hepatic protection is mainly mediated through its powerful antioxidative capacity, inhibition of hepatocyte apoptosis and suppression of inflammatory cytokines via signaling pathways^57,58^. Procyanidin B2 was investigated against hepatic oxidation in diabetic rats and exhibited a protective effect against CCl_4_-induced hepatic injury by elevating the antioxidative defense potential and consequently suppressing the inflammatory response^59,60^. The hyperoside and isoquercitrin in AVF could be potential natural hepatoprotective agents protecting the liver from injury though inhibition of oxidative stress as well as regulation of acetaminophen metabolism, and reports revealed that administration of these compounds effectively restored liver functions^39,61^. Although it has been speculated that the derivatives of hyperoside and isoquercitrin are biotransformed into corresponding glycosides^62^ or partly involved in the hepatoprotective function of AVF^39^, little research has been performed on their biological activities. In addition, as a phloroglucinol derivative, hyperforin, found in *Hypericum perforatum* L., provided protection against free radical-induced DNA damage based on its scavenging activity^63^; however, there is currently no related information on the hepatoprotective activity of hyperforin or allamandin.

**Figure 5:**
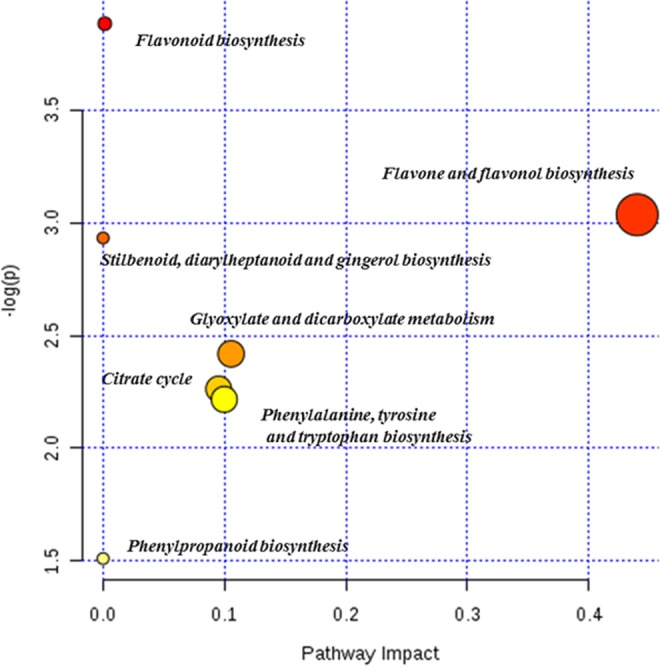
Pathway enrichment analysis showing the significantly changed biological pathways of AVF under salt stress.

The most significantly impacted biological pathways (Fig. 5 and Table S4), e.g., flavonoid biosynthesis and flavone and flavonol biosynthesis, were in line with our previous results^3^. In terms of the heat map, it seemed that a low concentration of salt stress could increase the accumulation of secondary metabolites, and AVF exposed to low levels of salt stress might be considered a source for dietary supplementation of polyphenols.

## Conclusion

In this study, fingerprint-activity relationship modeling was established to perform a quality evaluation of salt-tolerant AVF based on chemical fingerprints and efficacy. First, UFLC-Triple TOF-MS/MS fingerprints were established, and cluster analysis was performed based on the common characteristic peaks to evaluate the similarity between groups. Second, salt-stressed AVF protected against CCl_4_-induced acute liver injury in mice, and the effects of AVF were achieved via a reduction in serum transaminase activities, elevating hepatic antioxidative enzyme activities and ameliorating tissue lesions. Then, fingerprint-activity relationship modeling between the spectrum and the medicinal efficacy was examined by GCA and BCA to perform the quality evaluation and screen out bioactive markers for the AVF response to salt. According to the results, the abundant polyphenols might be responsible for the protective effects against acute liver damage in mice, and AVF with low-salt treatment was better in terms of efficacy than the other three groups. This strategy could serve as a useful reference for the quality evaluation, the discovery of bioactive markers and the future exploitation of salt-tolerant Chinese herbal medicines.

## Supplementary information

Supporting information
