# Supplementary material for: A strategy for quality evaluation of salt-treated Apocyni Veneti Folium and discovery of efficacy-associated markers by fingerprint-activity relationship modeling
Source: Sci Rep. 2019 Nov 13;9:16666. doi: 10.1038/s41598-019-52963-3 (PMC6853957; doi:10.1038/s41598-019-52963-3)
Supplement: Supplementary file 1 — Supporting information [file 41598_2019_52963_MOESM1_ESM.pdf]

Supplementary information

A strategy for quality evaluation of salt-treated Apocyni Veneti Folium and discovery of efficacy-associated markers by fingerprint-activity relationship modeling

Cuihua Chen[1,2], Jiali Chen[1], Jingjing Shi[1], Shuyu Chen[1], Hui Zhao[1], Ying Yan[1], Yucui Jiang[2], Ling Gu[2], Feiyan Chen[2] & Xunhong Liu[1,3,4]

[1] College of Pharmacy, Nanjing University of Chinese Medicine, Nanjing 210023, China;

[2] School of Basic Medicine, Nanjing University of Chinese Medicine, Nanjing 210023, China;

[3] Collaborative Innovation Center of Chinese Medicinal Resources Industrialization, Nanjing 210023, China

[4] National and Local Collaborative Engineering Center of Chinese Medicinal Resources Industrialization and Formulae Innovative Medicine, Nanjing 210023, China

Correspondence and requests for materials should be addressed to X.L. (email: liuxunh1959@163.com).

Experimental part of gray correlation analysis

The specific method of gray correlation analysis is as follows: Set X_0= (x_0 (1), x_0(2), …, x_0 (n)) as the sequence of system behavior characteristics.

as the sequence of system of associated factors.

The correlation coefficient is defined as follows:

$$\xi(x_0(k), x_i(k)) = \frac{\min_i \min_k |x_0(k) - x_i(k)| + \rho \max_i \max_k |x_0(k) - x_i(k)|}{|x_0(k) - x_i(k)| + \rho \max_i \max_k |x_0(k) - x_i(k)|} \tag{1}$$

The gray correlation grade is formulated as follows:

$$\xi(X_0, X_i) = \frac{1}{n} \sum_{k=1}^{n} \xi(x_0(k), x_i(k)) \tag{2}$$

Abbreviations: min-minimum; max-maximum; k is different batches of RC (k = 0, 1, 2, 3 … 10); X_i is the main characteristic component of the fingerprints of AVFE; ξ is the correlation coefficient between the sequence of system of associated factors and sequence of system behavior characteristics in the 12 samples; ρ is the distinctive coefficient lying between $0 < \rho < 1$, and it is generally set as 0.5.

Table S1. The treatments of CCl_4-induced acute liver damage in mice

Groups	C_{NaCl}(mM)	Administration(/10g/d, 2 w)	Molding (18 h)
Control	-	0.2 mL of 0.5% CMC-Na	10 mL/kg olive oil
Model	-	0.2 mL of 0.5% CMC-Na	
Silymarin	-	100 mg/kg	
AVF treated with different C_{NaCl}	0	0.2 g	10 mL/kg CCl_4 (0.3%)
	100	0.2 g	
	200	0.2 g	
	300	0.2 g	
	0	2 g	
	100	2 g	
	200	2 g	
	300	2 g	

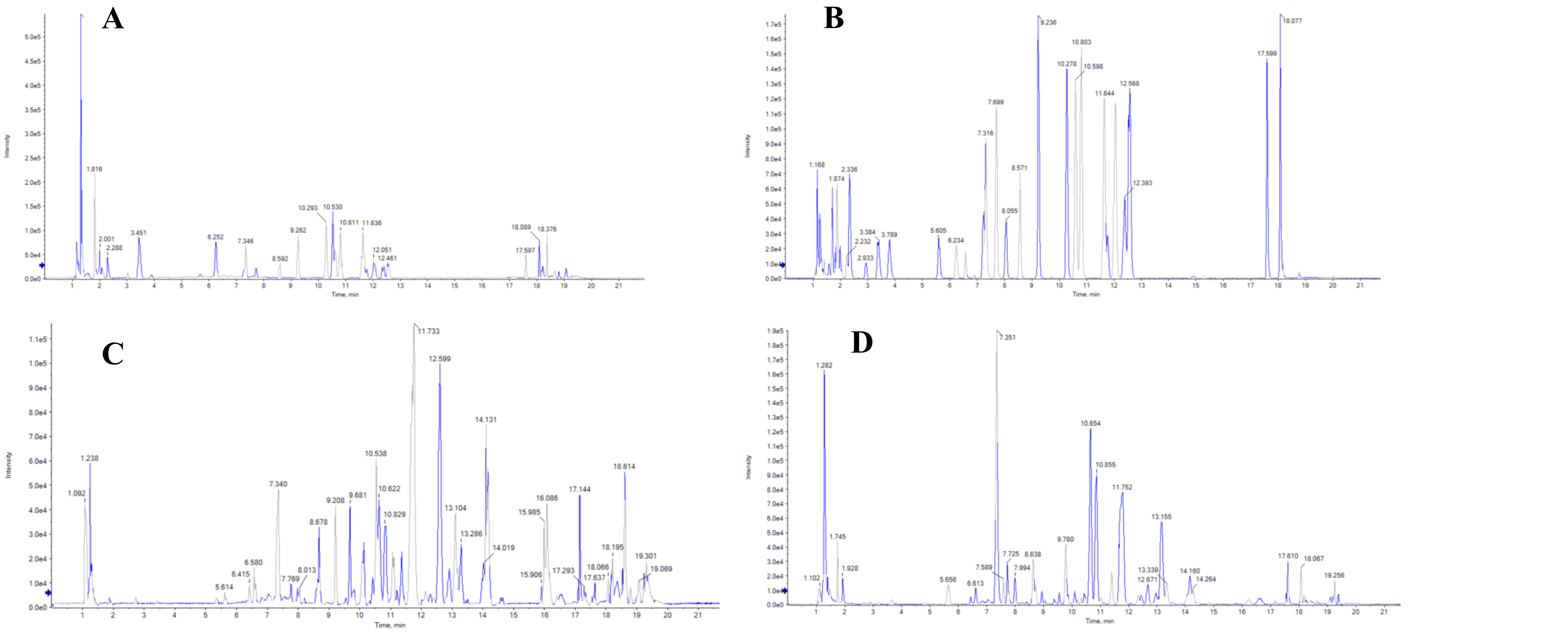

Figure S1. The representative UFLC-Triple TOF MS/MS base peak chromatogram of the reference mixtures (A and B) and AVF samples (C and D) under positive (A and C) and negative (B and D) ion modes.

Table S2. Relative peak area of each common peak from AVF samples based on UFLC-MS/MS under positive and negative ion modes.

Peak No.	retention time (t_R)	peak area of each common peak											
		S1	S2	S3	S4	S5	S6	S7	S8	S9	S10	S11	S12
P1	1.74	1281999	1318577	1283637	1497083	1330278	1361715	1016766	1292749	1173614	1293294	1305655	1096494
P2	7.34	2976496	3071094	2750043	2864994	3063018	2846549	3483566	2481065	2217402	3182102	2391824	3067139
P3	7.59	713022.1	556883.1	679318.6	634105.4	484328.5	647702.5	538847.9	498029.9	528486.9	606310.4	621754.1	491092.2
P4	7.75	591222.4	596551.1	493785	473637.5	579067.9	543643.9	591079	673860.8	593280	559335.2	481901.4	537417.2
P5	7.98	697747.8	812874.2	610518.9	613063.8	822623.3	822249.3	682402.2	687149.2	831263	835905.3	652230.4	712367.3
P6	8.70	158486.9	1069440	1121944	1584309	1333381	1603254	1123194	999028.2	1143681	1068847	1119576	1059546
P7	9.78	1767959	1727527	1558790	5962057	1592891	1704408	1610394	1603232	1526542	1740838	1685955	1633357
P8	10.63	3674779	3323271	3674536	3944927	3480629	3967399	4112088	3567420	3332172	4289482	3135121	4089885
P9	10.82	2596033	2599204	2817879	4507439	2225519	1913463	2303422	2283311	2178616	2167376	2687385	2535004
P10	12.39	396536.2	439030.7	441798	381578.6	434489.3	381072.7	422128.3	408417.8	448463.5	472366	465898.8	542102.3
P11	12.87	817510.4	726422.1	756789.1	687084.4	760234.5	674705.4	684571.5	745136.6	718447.8	652017.3	659005.5	710567.4
P12	18.77	5413441	5319590	8478688	7437396	8129842	7727643	5822897	7980594	9100006	8856182	12089240	9490518
N1	1.29	4132671	4222983	3990797	4808169	4089322	4013641	4812124	5043389	4032260	4569992	4757283	4089123
N2	6.43	180233.2	123278.1	95858.84	242573.3	227224.7	199010.3	396819.9	308726.7	106191.2	345721.9	366926.4	301318.7
N3	7.35	3171964	3749809	3507047	4963447	4186069	4170728	4329710	4540405	3144986	4430560	4471794	4256136
N4	7.59	331217.4	312730.3	288082.3	381934.5	366191.9	329614.2	464237.8	427752.7	188827.2	347840.3	370283.6	351101.9
N5	7.75	772343.1	758423.8	544822.8	809902.9	842094.2	953501	841736.1	768173.8	320099.5	753939.4	709375.6	861161.5
N6	7.99	849236.3	939723.9	668686.4	1132477	965402.1	1073498	1127839	1105088	591746.4	810186.3	883184	828938
N7	10.09	170234.7	169748.6	125384.4	259065	227770.5	305732.3	242489.4	204415	113918.2	286024.8	324601.3	301998.8
N8	10.62	2538494	3104897	2545921	2531206	2873730	2940794	3359603	3148339	1847988	2415497	2206509	2383883
N9	10.82	2658243	2780074	2225760	3012512	2439643	2587535	2641289	2360547	1571868	2087450	2068266	2111358
N10	11.38	1027519	1026518	687238	1123113	970402.7	934919	1298373	1012608	511654.9	864419.5	853410.1	1012895

N11	12.68	355055.8	412819.7	465828.5	415512.1	539073.3	472550.8	417678.8	410338.9	449155.4	361392	355061.6	325789.3
N12	17.18	252895.1	232685.8	285011.5	273161.2	299216.4	350077	354912	417803.5	277647.5	321863.3	367881.3	315974.2
N13	18.08	811458.1	755004.3	594752	796961.4	704543.1	519370.9	538282.9	614493.1	316044.5	594353.9	752847.1	678123.3
N14	19.25	594829.9	610905.1	326330	665215.1	695718	1010288	641864.7	447937.4	215822.3	484915.8	488391.5	594325.9

Table S3. Effects of AVF extract on the enzyme activities and hepatic MDA content after CCl_4 treatment in mice. Data are the mean ± SD (n = 6). Different letters (a, b, c, d, e, f and g) following values in the same row indicate significant differences among salt treatments using Duncan's multiple-range test at $p < 0.05$.

	ALT (U/L)	AST (U/L)	MDA (nmol/mg prot)	SOD (U/mg prot)	CAT (U/mg prot)	POD (U/mg prot)
Control	25.4±2.86f	24.63±4.12g	2.4±0.1e	472.7±15.15a	45.6±3.56a	0.73±0.05a
Model	130.67±8.57a	99.77±3.82a	5.43±0.42a	212.77±7.37g	25.1±2.36e	0.31±0.03e
Silymarin	54.63±7.84e	39.3±4.48f	3.5±0.17d	400.47±18.15c	40.1±1.11c	0.57±0.02b
L0	92.57±4.27c	68.7±3.9c	4.07±0.15c	237±9.17g	32.07±1.85d	0.35±0.02de
L100	96.83±3.04bc	68.37±5.3c	4.3±0.1c	284.27±13.23e	35.07±1.47d	0.39±0.02d
L200	103.27±4.2b	70±3.05bc	4.37±0.15bc	263.53±7.65f	34.47±1.58d	0.35±0.03de
L300	99.1±4.16bc	76.1±2.55b	4.63±0.15b	244.17±7.85f	34.13±1.01d	0.35±0.01e
H0	64.8±3.59d	52.97±3.3d	3.67±0.12d	372.13±12.4d	42.2±1.23bc	0.51±0.03c
H100	54.17±3.59d	40.93±2.84ef	3.4±0.1d	420.93±7.48b	44.1±1.75ab	0.55±0.03bc
H200	53.8±2.1e	46.6±0.92e	3.4±0.1d	412.27±8.55bc	39.4±1.25c	0.56±0.04bc
H300	72.23±3.63d	54.1±3.61d	3.7±0.17d	378.23±10.3d	39.9±1.9c	0.49±0.03d

Table S4. Analysis of disturbed biological pathways in AVF exposed to salt stress.

Pathway name	Match status	*p*	Impact
Flavonoid biosynthesis	2/43	0.020559	0.00137
Flavone and flavonol biosynthesis	1/9	0.048009	0.44
Stilbenoid, diarylheptanoid and gingerol biosynthesis	1/10	0.053219	0.0
Glyoxylate and dicarboxylate metabolism	1/17	0.089012	0.10544
Citrate cycle (TCA cycle)	1/20	0.10399	0.0952
Phenylalanine, tyrosine and tryptophan biosynthesis	1/21	0.10894	0.09982
Phenylpropanoid biosynthesis	1/45	0.22083	0.0

Match Status means the ration of matching number of biomarkers uploaded to the number of compounds in pathways; *p* value was calculated by pathway analysis; Impact value was calculated by topology analysis.
